# Prediction of severe hypertriglyceridemia-associated acute pancreatitis using a nomogram based on CT findings and blood biomarkers

**DOI:** 10.1097/MD.0000000000037911

**Published:** 2024-04-26

**Authors:** Jun Dong, Yuhang Shen, Zhihuai Wang, Jiankang Zhang, Xihu Qin, Chunfu Zhu, Yuan Gao, Qiang Yu

**Affiliations:** a Department of Hepato-biliary-pancreatic Surgery, The Affiliated Changzhou No. 2 People’s Hospital of Nanjing Medical University, Changzhou, China; b The Institute of Hepatobiliary and Pancreatic Diseases, The Affiliated Changzhou No. 2 People’s Hospital of Nanjing Medical University, Changzhou, China.

**Keywords:** blood biomarkers, hepatic steatosis, hypertriglyceridemia-associated acute pancreatitis, nomogram, severity

## Abstract

Hypertriglyceridemia is a common cause of acute pancreatitis (AP). Fatty liver, a manifestation of metabolic syndrome, is related to the severity of AP. The present study aimed to construct an accurate predictive model for severe AP (SAP) by combining the fatty liver infiltration on a computerized tomography (CT) scan with a series of blood biomarkers in patients with hypertriglyceridemia-associated AP (HTG-AP). A total of 213 patients diagnosed with HTG-AP were included in the present retrospective study. Clinical information and imageological findings were retrospectively analyzed. The model was constructed from independent risk factors using univariate analysis, the least absolute shrinkage and selection operator method. Subsequently, the data from the training group of 111 patients with HTG-AP was analyzed using logistic regression analysis. The efficacy of the model was verified using an external validation group of 102 patients through the receiver operating characteristic curve (ROC). Independent predictors, including serum calcium, C-reactive protein, lactate dehydrogenase and liver-to-spleen CT attenuation ratio (L/S ratio), were incorporated into the nomogram model for SAP in HTG-AP. The model achieved a sensitivity of 91.3% and a specificity of 88.6% in the training group. Compared with the Ranson model, the established nomogram model exhibited a better discriminative ability in the training group [area under the curve (AUC): 0.957] and external validation group (AUC: 0.930), as well as better calibration and clinical benefits. The present study demonstrates that the constructed nomogram based on CT findings and blood biomarkers is useful for the accurate prediction of SAP in HTG-AP.

## 1. Introduction

Acute pancreatitis (AP) is a common inflammatory disorder of the pancreas that causes severe abdominal pain and multiple organ dysfunction. The annual incidence of AP ranges from 13 to 45 per 100,000 individuals.^[1]^ Hypertriglyceridemia has become the second most common cause of AP,^[2]^ and related published studies have revealed that AP caused by hypertriglyceridemia [i.e., hypertriglyceridemia-associated AP (HTG-AP)] is more common than AP caused by other factors.^[3,4]^ The diagnosis of HTG-AP can be made when serum triglyceride (TG) levels are ≥ 11.3 mmol/L (1000 mg/dL) in the presence of AP; it has been recommended that serum TG levels ≥ 5.65 mmol/L (500 mg/dL) accompanied by milky serum can also be considered as a diagnostic criterion.^[5]^ HTG-AP has a higher morbidity due to complications and poorer outcomes than other types of AP.^[6,7]^ The Atlanta classification defines 3 degrees of severity: Mild acute pancreatitis (MAP), moderately severe acute pancreatitis and severe acute pancreatitis (SAP). SAP is defined by persistent organ failure, that is, organ failure > 48 h. Organ failure often manifests with respiratory, renal, or cardiovascular failure.^[8]^ Common stratification and prognostic models for AP include the Acute Physiology and Chronic Health Evaluation-II score (APACHE-II), Ranson score and BISAP score, as well as the Glasgow scoring system (GCS). The original Ranson criteria include a scoring system consisting of 11 parameters used to assess the severity of AP, which were initially used to score alcoholic pancreatitis. It includes inflammation indicators, body fluid loss, liver and kidney function indicators, but does not include lipid metabolism indicators. Similarly, other scores are not particularly applied in HTG-AP due to the specific etiology and pathophysiological process. On the one hand, these system scores are inaccurate in predicting the severity of HTG-AP and prognosis. On the other hand, they cannot be displayed graphically.^[9,10]^ Herein, more attention was paid to the Ranson score as it is the most widely used and has maintained its clinical effectiveness for years. In clinical practice, the data demonstrated that the imaging findings of the pancreas on an early computerized tomography (CT) scan could only reflect the severity at the time of admission, but could not predict the prognosis of HTG-AP. Therefore, more parameters from CT scans are required to anticipate the severity of HTG-AP in future therapy. In recent years, studies have found that abnormal lipid metabolism is the main cause of HTG-AP.^[11–14]^ However, in addition to lipid profiles, there is a lack of examinations to classify the abnormality of lipid metabolism vividly. The present study promotes a multidimensional judgment combined with laboratory analyses and imageology for the diagnosis and prognosis of metabolic diseases; this may be helpful for evaluating the prognosis of HTG-AP and may enable early intervention for further blocking the development of MAP to severe pancreatitis. Liver fatty infiltration on a CT scan was selected and combined with hematological indicators to grade the severity of HTG-AP at an early stage. This novel established system scoring can help doctors to assess and treat SAP at an early stage, as well as to reduce the incidence of SAP.

## 2. Methods

### 2.1. Study population

From January 2017 to December 2021, AP was diagnosed in 1732 patients at The Affiliated Changzhou No. 2 People’s Hospital of Nanjing Medical University. A total of 346 hospitalized patients who met the diagnostic criteria of HTG-AP were recruited in the present study, and 213 patients met the inclusion and exclusion criteria that were used to explore risk factors for severe HTG-AP.

The inclusion criteria were as follows: A diagnosis of HTG-AP; admission within 72 hours following onset; an age older than 18 years; and unenhanced abdominal CT and complete blood tests were performed within 48 hours of admission. The exclusion criteria were as follows: Patients who did not undergo serum lipid detection within 48 hours after treatment upon hospitalization; AP due to other etiologies (including gallstones, alcohol, autoimmune, drug-induced, pancreatic tumor-related etiology of AP); patients who received treatment at another hospital; and incomplete information available.

### 2.2. Classification and definitions

According to the grades of severity standard in the Revised Atlanta Classification, the patients were classified into the SAP groups and non-SAP groups, including the MAP group and moderately severe acute pancreatitis group.

HTG-AP was defined in patients with AP when the of serum level TG was: Over 1000 mg/dL; and between 500 and 1000 mg/dL with lactescent serum at the time of admission.

Organ failure was determined according to the improved Marshall score standard in the Revised Atlanta classification. Three organ systems were assessed to define organ failure: Respiratory, cardiovascular and renal. A score of 2 or more in any system defines the presence of organ failure.

### 2.3. Clinical information and image analysis

All laboratory data were collected within 48 hours of admission and the medical records and recorded the required data were reviewed, including clinical baseline characteristics, physiological indicators and laboratory indicators.

At the authors’ institution, an initial CT scan is usually performed in all patients with AP within 48 hours after admission, regardless of the severity of AP. Unenhanced phase CT images were retrospectively reviewed by a radiologist blinded to clinical data. We measured the mean Housefield units (HU) for regions of interest (ROIs) in the liver and spleen. At the same slice, 4 ROIs were placed in the left medial segment, left lateral segment, right anterior segment and right posterior segment of the liver, respectively, and one ROI was placed in the spleen. ROIs ranged from 200 to 300 mm^2^. The liver-to-spleen attenuation ratio (L/S ratio) was calculated by dividing the mean hepatic HU by the splenic HU.

### 2.4. Statistical analysis

Statistical analysis was performed using R software and SPSS 26.0 (IBM Corp., USA). The continuous variables were compared using a *t* test for normally distributed data or the Mann–Whitney *U* test for non-normally distributed data. The categorical variables were compared using the χ^2^ test or Fisher’s exact test and Spearman’s correlation was used to evaluate the correlation between variables. The least absolute shrinkage and selection operator (LASSO) method, which is suitable for the reduction in high-dimensional data, was used to select the optimal predictive features in risk factors from the patients with HTG-AP. The factors exhibiting a significant correlation (*P* < .05) were screened out for use in subsequent multivariate logistic regression analysis. Factors with a *P* value < .05 in the aforementioned analysis were identified as independent factors. According to the cutoff values, factors were used in the three-way classification, and based on the *β* coefficient of each factor; the prediction model was shown by the nomogram. The performance of the model was subsequently tested in the independent validation group using the formula and cutoff values derived from the training group. Receiver operating characteristic (ROC) curve was used to evaluate the diagnostic performance of the nomogram, and the calibration curve was plotted to assess the calibration of the nomogram using the Hosmer-Lemeshow test. Decision curve analysis (DCA) was performed to calculate the net benefit from the application of the nomogram at different threshold probabilities. *P* < .05 was considered to indicate a statistically significant difference.

## 3. Results

### 3.1. Baseline characteristics and representative cases

A total of 213 patients were diagnosed with HTG-AP during the study period, who underwent an initial blood biomarker examination and CT scanning upon admission. According to the order of the hospitalization, 111 participants were included in the training group and 102 participants were included in the validation group. The baseline characteristics of the study participants are presented in Table 1. The median age of the patients in the training group was 40 years and that of the patients in the validation group was 37.5 years. There were 82 male patients (72.8%) in the training group and 70 (68.7%) in the validation group. A total of 75 patients (67.5%) in the training group and 63 patients (61.8%) in the validation group had chronic diseases (hypertension or diabetes). The median body mass index values of the patients in the training and validation groups were 27.9 and 27.4, respectively. There were no significant differences in the general variables between the 2 groups. Grouping in chronological order was set for further model construction and validation.

**Table 1:** Baseline clinical information of 213 patients included in this study.

Variables	Training (n = 111)	Validation (n = 102)	t/Z/χ^2^	*P*
Age	40 (33, 47)	37.5 (32, 47)	*Z* = −1.031	.301
Gender, man	82 (72.8%)	70 (68.7%)	χ^2^* = *1.112	.292
Chronic diseases	75 (67.6%)	63 (61.8%)	χ^2^* = *1.159	.282
BMI	27.9 (26, 30)	27.35 (24.12, 29.45)	*Z* = −1.920	.055
WBC	12.63 ± 4.06	12.51 ± 4.21	*t* = 0.219	.857
HCT	43.44 ± 5.64	42.92 ± 5.35	*t* = 0.685	.494
RDW	12.9 (12.4,13.4)	12.8 (12.28, 13.43)	*Z* = −0.957	.339
MPV	10.99 ± 1.17	10.74 ± 0.97	*t* = 1.744	.083
PDW	13.8 (11.6, 15.8)	13.2 (11.6, 14.9)	*Z* = −0.766	.444
Tbil	14 (10.3, 20.6)	14.8 (11.6, 21.4)	*Z* = −0.973	.331
TP	63.92 ± 8.04	63.29 ± 7.72	*t* = 0.575	.566
ALB	39.1 (36, 41.7)	37.5 (34.7, 41.0)	*Z* = −1.764	.078
ALT	20.8 (12.3, 27.8)	21.2 (14.9, 35.2)	*Z* = −1.549	.121
AST	21.9 (15.9, 32.0)	22.4 (16.9, 31.0)	*Z* = −0.147	.883
Glu	12.27 ± 4.93	12.82 ± 4.73	*t* = −0.961	.336
TCH	8.8 (6.7, 12.3)	8.9 (6.9, 10.7)	*Z* = −0.250	.802
TG	13.83 (8.48, 20.48)	14.7 (10.3, 18.0)	*Z* = −0.559	.576
Ca	2.1 (1.94, 2.24)	2.1 (1.89, 2.19)	*Z* = −1.142	.254
Cr	61 (49, 78)	60.6 (51.4, 74.1)	*Z* = −0.457	.647
BUN	4.2 (3.1, 5.2)	3.8 (2.9, 5.2)	*Z* = −1.287	.198
LDH	250 (202, 344)	277.50 (201.75, 393.25)	*Z* = −0.950	.342
D-Dimer	1.11 (0.41, 2.3)	1.035 (0.428, 3.073)	*Z* = −0.383	.702
PT	12.1 (11.4, 13)	11.8 (11.18, 12.73)	*Z* = −1.791	.073
PCT	0.166 (0.060, 0.676)	0.13 (0.05, 0.765)	*Z* = −0.306	.759
CRP	67.71 (25.3, 151.93)	77.15 (14.20, 163.10)	*Z* = −0.022	.982
L/S	0.76 (0.57, 0.97)	0.83 (0.62, 0.97)	*Z* = −0.536	.592

ALB = albumin, g/L, ALT = alanine aminotransferase, U/L, AST = aspartate transaminase, U/L, BMI = body mass index, BUN = blood urea nitrogen, mmol/L, Ca = serum calcium, mmol/L, Cr = creatinine, μmmol/L, CRP = C-reactive protein, mg/L, Glu = glucose, mmol/L, HCT = hematocrit, %;, LDH = lactate dehydrogenase, U/L, MPV = mean platelet volume, fL, PCT = serum procalcitonin, ng/mL, PDW = platelet distribution width, %, PT = prothrombin time, s, RDW = red blood cell distribution width, %, Tbil = total bilirubin, μmmol/L, TCH = total cholesterol, mmol/L, TG = triglyceride, mmol/L, TP = total protein, g/L, WBC = white blood cell, ×10^9^/L.

**Figure 1.**
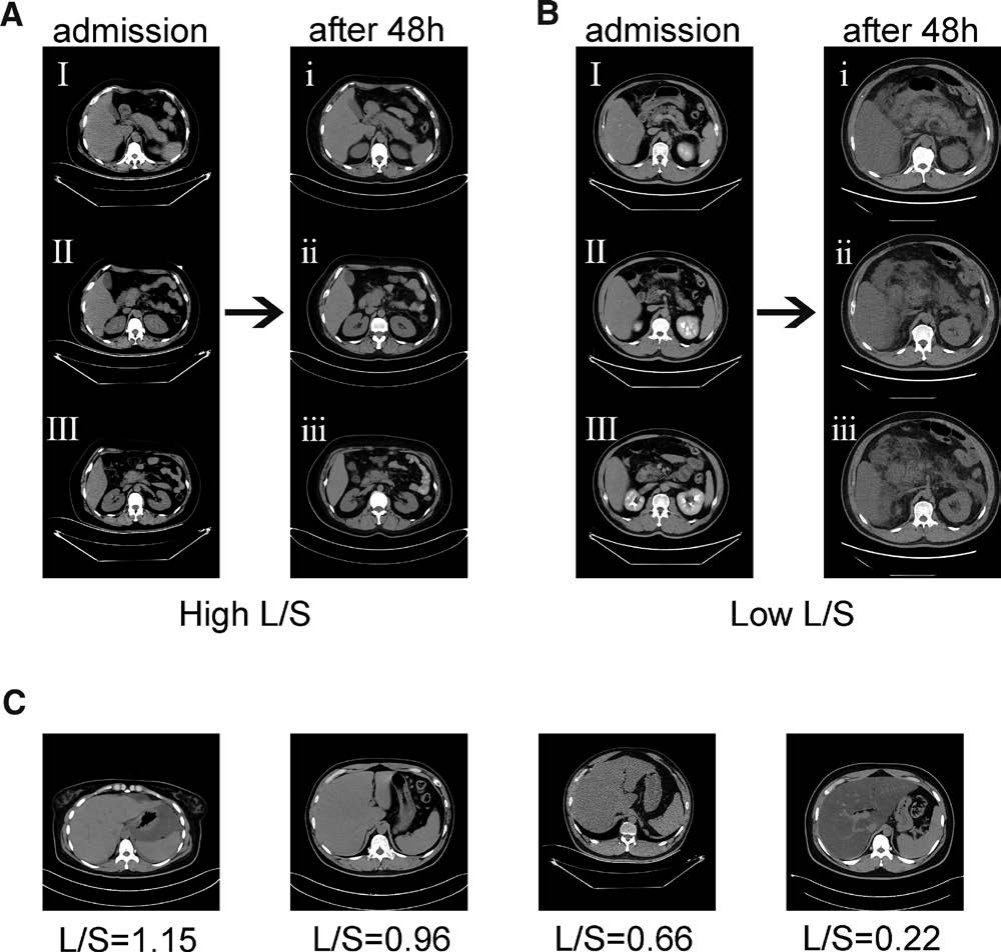
Representative cases of HTG-AP on CT scans. (A) Changes in pancreatic CT findings in patients with a high L/S ratio. (B) Changes in pancreatic CT findings in patients with a low L/S ratio. (C) CT scans of varying degrees of fatty liver in patients with HTG-AP. I and i, body and tail of pancreas; II and ii, head of pancreas; III and iii, uncinate process of pancreas.

In the training group, the L/S ratio obtained from a CT scan of the patients who were admitted at an early stage revealed significant differences. However, no differences were found in the CT findings of the pancreas and peripancreatic tissue, which only revealed pancreatic enlargement. However, 48 hours later, the CT findings of the patients with a low L/S ratio revealed evident peripancreatic exudation, while the CT findings of the patients with a high L/S ratio did not differ significantly from those at the time of admission (Fig. 1A and B).

The study participants were then categorized into 3 groups according to their L/S ratio (from a lower to a higher ratio). Those with a lower ratio were categorized as having mild fatty liver, followed by the second category which was moderate fatty liver, and the last category which was severe fatty liver. Representative CT images of the varying degrees of fatty liver are presented in Figure 1C.

### 3.2. Risk factors for severe acute pancreatitis in HTG-AP

**Figure 2.**
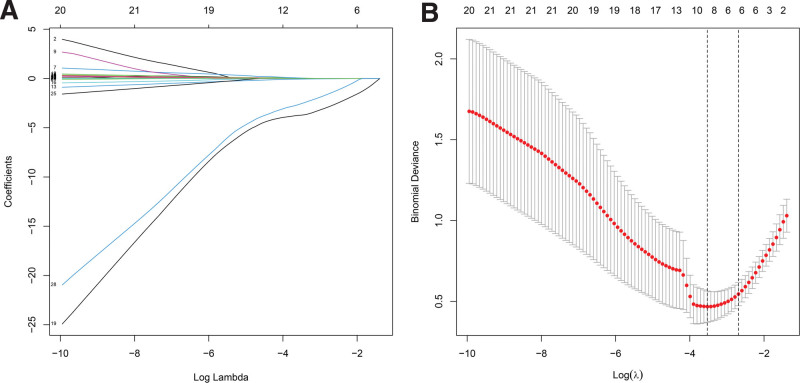
Penalty chart of predictive factors for severity in patients with HTG-AP. (A) LASSO coefficient curve of 28 variables. The coefficient distribution map is generated relative to the logarithmic (λ) sequence. (B) Ten-times cross-validation was used in the LASSO regression, and the binomial deviation is plotted against the logarithm (λ). The dashed vertical line is drawn with the best value using the minimum criteria (left dashed line) and the 1 standard error criterion (right dashed line). In the present study, the predictors were selected according to the minimum criteria, where the optimal λ leads to 9 non-zero coefficients.

In the training group, univariate analysis (Table 2) revealed that patients with SAP had a higher white blood cell count (*P* = .03), higher hematocrit level (*P* = .002), elevated coefficient variation of red blood cell distribution width (*P* = .002), higher mean platelet volume (*P* = .01) and platelet distribution width level (*P* = .004) in the blood routine examination compared with those without SAP. In the blood biochemical markers, the patients with SAP had lower total protein (TP) (*P* < .001), albumin (*P* < .001) and serum calcium (*P* < .001) levels, but higher aspartate aminotransferase (*P* = .001), glucose (*P* = .015), creatinine (*P* < .001), blood urea nitrogen (*P* < .001), lactate dehydrogenase (LDH) (*P* < .001), serum procalcitonin (PCT) (*P* < .001) and C-reactive protein (CRP) (*P* < .001) levels. In comparison to the patients without SAP, those with SAP had a longer prothrombin time (*P* = .019) and higher D-dimer levels (*P* < .001). In addition, the patients with SAP had a lower L/S ratio (*P* < .001), indicating that had more severe hepatic steatosis. The present study then examined the correlation between all factors and the outcomes. Spearman’s correlation analysis (Table 2) revealed that all factors, apart from white blood cell count significantly correlated with the outcomes of HTG-AP in the univariate analysis. The variables were further screened in the LASSO regression to construct a simple and efficient prediction model. Including albumin, serum calcium, serum creatinine, LDH, CRP and the L/S ratio, 6 potential predictors were extracted from the 28 factors based on the training group and were with non-zero coefficients in the LASSO regression (Fig. 2A and B). Based on the aforementioned results, the model was established using multivariate logistic regression and represented by a nomogram. The results revealed that 5 variables were incorporated into the equation after 5 steps of screening, of which serum calcium (*P* = .04), LDH (*P* = .009), CRP (*P* = .016) and the L/S ratio (*P* = .005) were independent predictors of SAP in patients with HTG-AP (Table 3).

**Table 2:** Univariate analysis and Spearman correlation analysis in the training group.

Variables	Univariate analysis		Spearman correlation	
	*t/Z/* χ^2^	*P*	*r*	*P*
Age	*t* = 0.008	.993	0.038	.693
BMI	*t* = 1.968	.052	0.210	.027
Gender	χ^2^ = 0.471	.493	−0.051	.595
Chronic diseases	χ^2^ = 0.081	.776	−0.022	.820
WBC	*t* = 2.197	.03	0.184	.053
HCT	*t* = 3.106	.002	0.261	.006
RDW	*t* = 3.256	.002	0.338	<.001
MPV	*t* = 2.627	.01	0.258	.006
PDW	*Z* = −2.875	.004	0.274	.004
Tbil	*Z* = −0.415	.678	0.04	.680
TP	*Z* = −3.569	<.001	−0.340	<.001
ALB	*Z* = −4.948	<.001	−0.472	<.001
ALT	*Z* = −0.218	.827	−0.021	.828
AST	*Z* = −3.191	.001	0.304	.001
Glu	*Z* = −2.437	.015	0.232	.014
TCH	*Z* = −0.822	.411	0.078	.413
TG	*Z* = −1.393	.164	0.133	.165
Ca	*Z* = −5.742	<.001	−0.547	<.001
Cr	*Z* = −4.206	<.001	0.401	<.001
BUN	*Z* = −3.764	<.001	0.359	<.001
LDH	*Z* = −5.992	<.001	0.571	<.001
D-Dimer	*Z* = −4.224	<.001	0.403	<.001
PT	*Z* = −2.345	.019	0.224	.018
PCT	*Z* = −6.735	<.001	0.642	<.001
CRP	*Z* = −4.264	<.001	0.407	<.001
L/S	*t* = 5.122	<.001	−0.418	<.001

ALB = albumin, g/L, ALT = alanine aminotransferase, U/L, AST = aspartate transaminase, U/L, BMI = body mass index, BUN = blood urea nitrogen, mmol/L, Ca = serum calcium, mmol/L, Cr = creatinine, μmmol/L, CRP = C-reactive protein, mg/L, Glu = glucose, mmol/L, HCT = hematocrit, %, LDH = lactate dehydrogenase, U/L, MPV = mean platelet volume, fL, PCT = serum procalcitonin, ng/mL, PDW = platelet distribution width, %, PT = , prothrombin time, s, RDW = red blood cell distribution width, %, Tbil = total bilirubin, μmmol/L, TCH = total cholesterol, mmol/L, TG = triglyceride, mmol/L, TP = total protein, g/L, WBC = white blood cell, ×10^9^/L

**Table 3:** Multivariate logistic regression model in the training group.

Variable	OR (95% CI)	*P*
ALB	0.808 (0.626–1.045)	.101
Ca	0.692 (0.486–0.984)	.040
LDH	1.008 (1.002–1.015)	.009
CRP	1.014 (1.003–1.026)	.016
L/S	0.677 (0.514–0.891)	.005

ALB = albumin, g/L, Ca = serum calcium, mmol/L, CI = confidence interval, CRP = C-reactive protein, mg/L, LDH = lactate dehydrogenase, U/L, OR = odds ratio.

### 3.3. Development and validation of the prediction nomogram

Based on the ROC curve analysis (Table 4), the optimal cutoff values for serum calcium, LDH, CRP and the L/S ratio were 1.95 mmol/L, 315 U/L, 122 mg/L and 0.64, respectively. The upper limit of normal for LDH and CRP was 245 U/L and 8 mg/L, while the lower limit of normal for serum calcium was 2.25 mmol/L. On the basis of previous studies and guidelines, 0.64 and 1 were defined as cutoff points in the three-way classification.^[7,15–17]^ Thus, the cutoff values and the limits of normal for the 4 factors were used in the three-way classification.

**Table 4:** Multivariate regression model parameters.

Variables	Multivariate analysis		ROC analysis		
	β	OR (95% CI)	*P*	AUC	*cutoff*
Ca	1.900	6.689 (1.467–30.508)	.014	0.890	1.95
LDH	1.321	3.748 (1.169–12.015)	.026	0.907	315
CRP	1.800	6.051 (1.572–23.289)	.009	0.790	122
L/S	1.268	3.553 (1.021–12.370)	.046	0.797	0.64

AUC = area under the curve, Ca = serum calcium, mmol/L, CI = confidence interval, CRP = C-reactive protein, mg/L, LDH = lactate dehydrogenase, U/L, OR = odds ratio.

**Figure 3.**
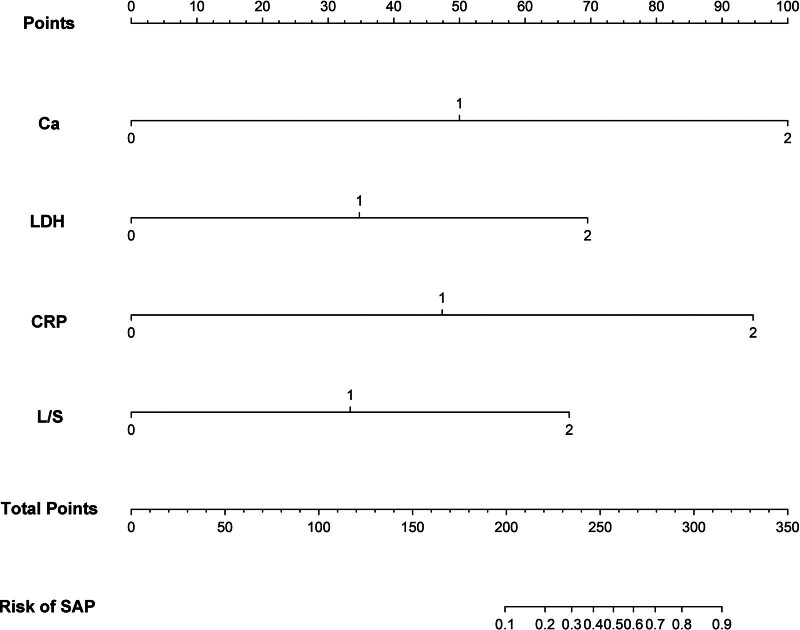
Nomogram of independent predictors of SAP in patients with HTG-AP. Ca (0, >2.25 mmol/L; 1, ≥1.95 and ≤ 2.25 mmol/L; 2, <1.95 mmol/L), LDH (0, <245 U/L; 1, ≥245 and ≤ 315 U/L; 2, >315 U/L), CRP (0, <8 mg/L; 1, ≥8 and ≤ 122 mg/L; 2, >122 mg/L), and L/S ratio (0, ≥1; 1, ≥0.64 and < 1; 2, <0.64).

Based on the β coefficient of each factor, a nomogram model was constructed, incorporating predictive factors to predict the risk of SAP in patients with HTG-AP (Table 4). The nomogram is illustrated in Figure 3 for serum calcium (0, >2.25 mmol/L; 1, ≥1.95 and ≤ 2.25 mmol/L; 2, <1.95 mmol/L), LDH (0, <245 U/L; 1, ≥245 and ≤ 315 U/L; 2, >315 U/L), CRP (0, <8 mg/L; 1, ≥8 and ≤ 122 mg/L; 2, >122 mg/L) and L/S ratio (0, ≥ 1; 1, ≥0.64 and < 1; 2, <0.64).

**Figure 4.**
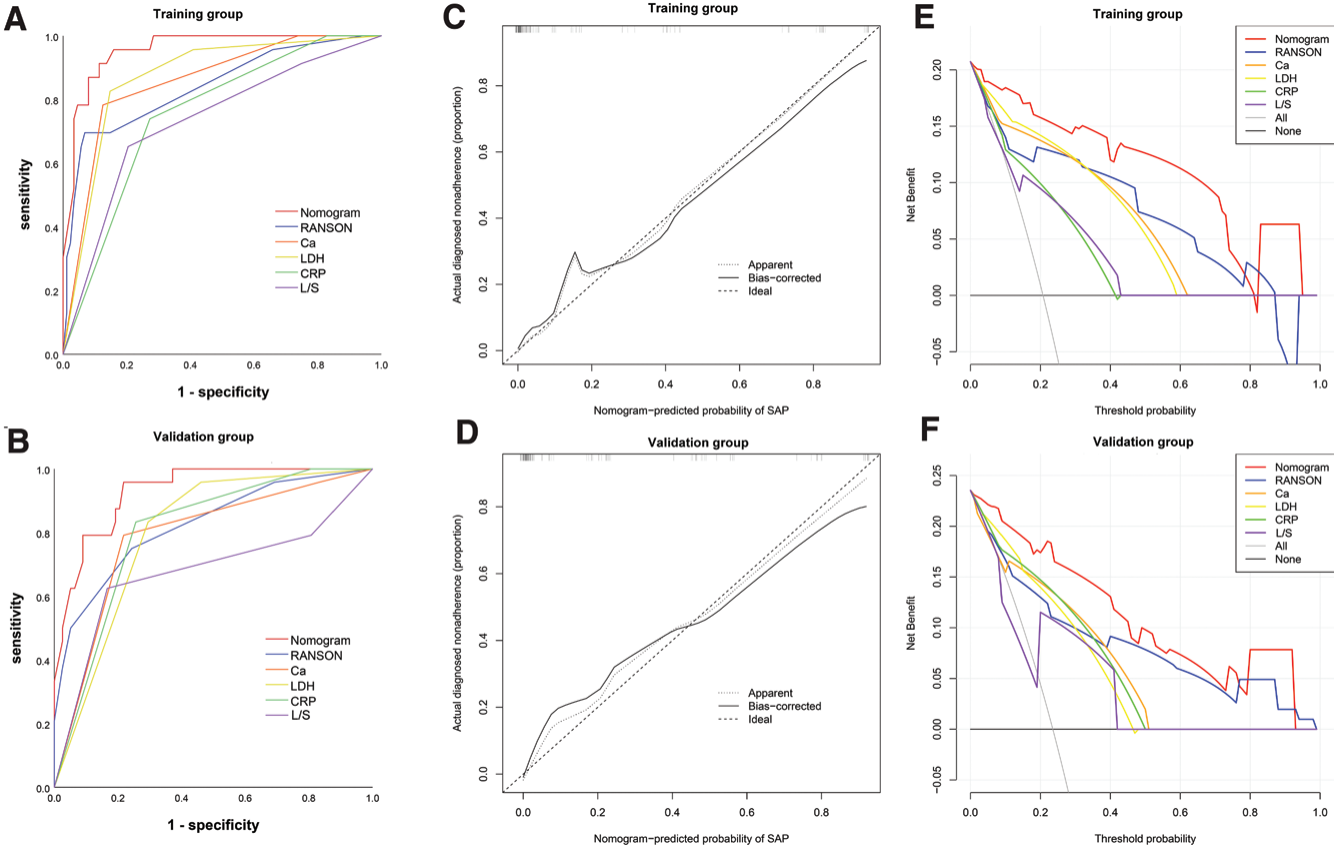
Assessment and validation of the nomogram. (A) and (B) Comparison of ROC curves between the nomogram model, Ranson model, serum calcium, LDH, CRP and L/S ratio in the training and validation groups; (C) and (D) Calibration curves of the nomogram in the training and validation groups; (E) and (F) Decision curve analysis for the nomogram in the training and validation groups.

The nomogram achieved a sensitivity of 91.3%, and a specificity of 88.6% in the training group. The ROC curve was plotted to compare the discrimination ability of the nomogram with the Ranson score and each factor alone. As shown in Figure 4A and B, the nomogram obtained the optimal discriminative ability with an area under the curve (AUC) value of 0.957 (95% confidence interval [CI]: 0.921, 0.993) in the training group. In the external validation cohort, the model also yielded the highest AUC value of 0.930 (95% CI: 0.880, 0.979) compared with the Ranson score (AUC: 0.823; *P* < .001), serum calcium (AUC: 0.788; *P* < .001), LDH (AUC: 0.799; *P* < .001), CRP (AUC: 0.804; *P* < .001), and L/S ratio (AUC: 0.678; *P* = .008).

The calibration curve of the nomogram achieved excellent agreement between the predicted and actual risk of SAP in the training group. The *P* value of the Hosmer-Lemeshow test was 0.986, suggesting no deviation from the good fit. The favorable calibration was further confirmed in the external validation group with a *P* value of .260 (Hosmer-Lemeshow test) (Fig. 4c and 4d).

To evaluate the clinical benefits provided by the prediction models, the DCAs for the nomogram, Ranson, serum calcium, LDH, CRP and L/S ratio are presented in Figure 4E and F. Across the reasonable threshold probability ranges in both the training and validation groups, DCA graphically demonstrated that the nomogram model demonstrated marked improvements compared with the Ranson, serum calcium, LDH, CRP and L/S ratio.

## 4. Discussion

Although the initial presentation of HTG-AP is similar to that of AP induced by other pathogeneses, hypertriglyceridemia aggravates the severity and related complications of AP.^[18,19]^ Thus, exploring the risk factors for severe HTG-AP in the early stages is critical for triaging patients appropriately to intensive care units and providing specific treatments.

The present study first screened potential SAP-related factors through the univariate analysis and Spearman’s correlation analysis. The independent predictors were then identified by LASSO regression and multivariate logistic regression, which was characterized by variable selection and regularization, while fitting generalized linear models to avoid overfitting. This approach that was applied to construct the model could improve the accuracies of estimation and prediction. In addition, each factor was classified into 3 levels according to their optimal cutoff values in the ROC curve analysis, which made the model more intuitive and convenient for application. To increase the reliability of the model, an external validation group was also used to assess the model. Finally, serum calcium, LDH and CRP levels, and the L/S ratio were confirmed as independent predictors for the risk of SAP and were integrated into the nomogram model. Subsequently, the model achieved a significantly improved diagnostic performance and provided more clinical benefits when comparing the ROC curves, calibration and DCA curves with those in the Ranson model, serum calcium, LDH, CRP and L/S ratio. The present study first established the prediction system in a nomogram that integrated the CT features of hepatic steatosis and blood biomarkers in patients with HTG-AP. This model may allow doctors to make decisions dependent on more than existing models which were not constructed specifically for HTG-AP.

Similar to previous reports that the average age of patients with HTG-AP is lower than that of patients with other types of AP, the present study revealed that the median age of the patients was 37.5 to 40 years.^[3,4,20]^ The prevalence rate of hypertension or diabetes was higher in patients with HTG-AP, including values of 67.5% and 61.8% in the training and validation groups, respectively. In addition, it was found that they had a high median body mass index (27.9 and 27.4). Patients with HTG-AP often have other complications, including metabolic diseases such as diabetes mellitus and obesity, and those with type 2 diabetes mellitus have an elevated risk of developing AP compared with patients without diabetes.^[21]^ Serum calcium, LDH and CRP levels, which were included factors in the final model, have been reported as risk factors for SAP in a number of previous studies.^[22–25]^ It is worth noting that although several factors were not included in the model that was ultimately constructed, they were also valuable in assessing the severity of AP. The results demonstrated that the D-dimer levels correlated with the severity of HTG-AP (Spearman’s correlation, *r* = 0.403). D-dimer is a specific product of degradation of cross-linked fibrin, which indirectly reflects the coagulation disorder. Accumulating evidence suggests that the hemostatic balance is impaired in patients with metabolic syndrome, including hypertriglyceridemia,^[26,27]^ which may be related to the increasing level of plasma protein factor XII (FXII), endothelial dysfunction,^[28]^ and the activation of platelets and the coagulation pathway by increased concentrations of very-low-density lipoprotein.^[29]^ In recent years, the diagnostic value of PCT has attracted increasing attention in various diseases, and the present study found that it was closely related to the severity of HTG-AP (Spearman’s correlation, *r* = 0.642). PCT can differentiate bacterial sepsis from a systemic inflammatory response, which could lead to the guidance of antibiotic use without an adverse effect on the outcome of acute pancreatitis.^[30]^ Therefore, PCT-guided care should be incorporated into future guidelines on the management of AP.

The present study demonstrated an association between HTG-AP and the L/S ratio (Spearman’s correlation, *r* = −0.418), which could reflect the degree of fatty liver. Consistent with the conclusions of other researchers,^[31–34]^ the incidence rates of SAP exhibited an upward trend with the aggravation of hepatic steatosis. As a key risk factor for fatty liver, HTG indicates an elevation of lipids in the blood, which are hydrolyzed to generate free fatty acids. The high levels of free fatty acids induced by HTG could result in inflammation and pancreatic tissue damage, and may thus exacerbate the development of pancreatitis.^[35]^ The activation of Kupffer cells by liver lipid infiltration leads to the expression of various inflammatory factors and simultaneously contributes to the inflammatory cascade.^[36]^ In addition, a previous study reported that nonalcoholic fatty liver disease aggravates AP by increasing bacterial translocation in the liver and pancreas.^[37]^ This finding may explain the reason for the association between the severity of HTG-AP and PCT in the present study, which is a sensitive indicator of bacterial infection. Therefore, the appropriate treatment of fatty liver and the improvement of lipid metabolism may contribute to the prognosis of pancreatitis in patients with HTG-AP.

The present study had several limitations. First, the progress of model construction in the training group was retrospective in nature, for which reason, inherent selection biases could be avoided. Furthermore, the present study was a single-center study and the study population was not sufficient in size. Therefore, further research and verifications are required in the future. Second, the present study could not confirm the severity of fatty liver by pathological examinations, although previous studies have demonstrated that the imaging and pathological examinations of fatty liver are in good agreement.

## Author contributions

**Conceptualization:** Jun Dong, Yuhang Shen, Yuan Gao, Qiang Yu.

**Data curation:** Jun Dong, Yuhang Shen, Qiang Yu.

**Formal analysis:** Jun Dong, Yuhang Shen, Qiang Yu.

**Funding acquisition:** Yuan Gao, Qiang Yu.

**Investigation:** Jun Dong, Yuhang Shen.

**Methodology:** Jun Dong, Qiang Yu.

**Project administration:** Jun Dong, Qiang Yu.

**Resources:** Jun Dong, Qiang Yu.

**Software:** Jun Dong, Yuhang Shen, Jiankang Zhang, Qiang Yu.

**Supervision:** Zhihuai Wang, Xihu Qin, Chunfu Zhu, Yuan Gao, Qiang Yu.

**Validation:** Jun Dong, Yuhang Shen, Zhihuai Wang, Jiankang Zhang, Yuan Gao, Qiang Yu.

**Visualization:** Jun Dong, Yuhang Shen.

**Writing – original draft:** Jun Dong, Yuhang Shen, Yuan Gao, Qiang Yu.

**Writing – review & editing:** Jun Dong, Yuhang Shen, Zhihuai Wang, Jiankang Zhang, Xihu Qin, Chunfu Zhu, Yuan Gao, Qiang Yu.
